# SARS-CoV-2 Antibodies in Commercial Immunoglobulin Products Show Markedly Reduced Cross-reactivities Against Omicron Variants

**DOI:** 10.1007/s10875-023-01486-8

**Published:** 2023-04-14

**Authors:** Hannes Lindahl, Puran Chen, Mikael Åberg, Hans-Gustaf Ljunggren, Marcus Buggert, Soo Aleman, C. I. Edvard Smith, Peter Bergman

**Affiliations:** 1 Department of Clinical Immunology and Transfusion Medicine, Karolinska University Hospital, Stockholm, Sweden; 2 Department of Clinical Neuroscience, Karolinska Institutet, Stockholm, Sweden; 3 Department of Medicine Huddinge, Karolinska Institutet, Stockholm, Sweden; 4 Department of Medical Sciences, Clinical Chemistry and SciLifeLab, Uppsala University, Uppsala, Sweden; 5 Department of Infectious Diseases, Karolinska University Hospital, Stockholm, Sweden; 6 Department of Laboratory Medicine, Karolinska Institutet, Stockholm, Sweden

**Keywords:** Primary immunodeficiency, Immunoglobulin replacement therapy, SARS-CoV-2, Omicron, Passive immunity, X-linked agammaglobulinemia

## Abstract

**Purpose:**

Patients with antibody deficiencies often receive maintenance treatment with donor plasma-derived immunoglobulin (Ig) preparations to decrease the incidence and severity of infections. We have previously shown that IgG antibodies to the original SARS-CoV-2 strain were not consistently present in off-the-shelf Ig batches produced up to approximately 18 months after the first identified case of COVID-19 in the USA and that Ig batches with anti-SARS-CoV-2 IgG primarily contained vaccine-induced spike specific antibodies. This study aimed to investigate the degree of cross-reactivity between vaccine-induced anti-SARS-CoV-2 antibodies against Wuhan strain and subsequent viral variants.

**Methods:**

Samples were collected from 74 Ig batches supplied by three different commercial manufacturers. All batches were used at the Immunodeficiency Unit at the Karolinska University Hospital from the start of the SARS-CoV-2 pandemic until September 2022. Antibody quantity and potential to neutralize virus entry into host cells were assessed against the original SARS-CoV-2 Wuhan strain and the following nine variants: Alpha, Beta, Delta, IHU, and the Omicron BA.1, BA.1.1, BA.1 with spike mutation L452R, BA.2, and BA.3.

**Results:**

Ig batches produced approximately 18 months after the SARS-CoV-2 outbreak (from around July 2021) and later consistently contained high quantities of antibodies that bind the Wuhan strain. The Ig batches had overall low reactivity to the SARS-CoV-2 nucleocapsid, which implies that plasma donor spike IgG essentially is the result of vaccination. We assessed the degree of cross-reactivity towards each virus variant by plotting the variant/Wuhan strain ratio, which was consistent regardless of production date, suggesting cross-reactivity with vaccine-induced antibodies rather than virus exposure in the plasma donor population. Viral variants that emerged later during the pandemic systematically had a lower reactivity ratio, except for the Delta and IHU variants. The Ig batches displayed markedly low neutralizing potential towards the Beta variant and all tested Omicron variants.

**Conclusion:**

Commercial Ig batches currently contain large quantities of SARS-CoV-2 vaccine-induced antibodies. Cross-reactivity with variant strains is evident but varies, with markedly low neutralizing potential observed against Omicron variants.

**Supplementary Information:**

The online version contains supplementary material available at 10.1007/s10875-023-01486-8.

## Introduction

Patients with immunoglobulin (Ig)G deficiency often receive prophylactic Ig replacement therapy (IgRT) to decrease the frequency and consequences of infections, primarily of the respiratory tract [1]. Donor-derived plasma is used to manufacture these Ig products. Each batch typically contains IgG from at least one thousand healthy individuals and therefore is representative of the serostatus of the donor population at the time of donation. Importantly, there is a variable delay between plasma donation and accessibility for use in the clinic. Moreover, any correlation between company and donor population is not predictable because plasma is sold on an international market. Hence, prediction of when immunity for an emerging pathogen will be reflected in commercially available IgG preparations can be difficult.

The rapid development and dissemination of SARS-CoV-2 vaccines have limited the severity of COVID-19. Vaccines have merely focused on the ancestral Wuhan-strain spike protein as the antigen. However, soon after SARS-CoV-2 vaccination was distributed globally, new variants with spike protein mutations emerged, leading to increased transmissibility and diminished vaccine efficacy against infection [2]. Although the vaccines are still effective against severe disease, immunodeficient persons possess increased mortality compared to immunocompetent persons after adjusting for demographic and clinical factors [3]. Although it has not been unequivocally shown that treatment with donor-derived polyclonal antibodies upon infection protects immunodeficient persons from severe COVID-19, some reports suggest efficacy [4–6]. Importantly, less is known regarding how standard prophylactic IgRT affects COVID-19 susceptibility. However, as with SARS-CoV-2 vaccines and monoclonal therapies, the potential efficacy of donor-derived antibodies is likely to wane as the virus evolves and new strains emerge and become dominant.

We have previously shown that antibodies to the original SARS-CoV-2 strain were not consistently present in off-the-shelf Ig batches produced up to approximately 18 months after the first identified case of COVID-19 in the USA [7]. Moreover, we observed that Ig batches with high quantities of SARS-CoV-2 antibodies primarily contained vaccine-induced antibodies. In the former report, we concluded that continued vaccination would raise the SARS-CoV-2 antibody content in the general population. This may improve the protective capacity of IgRT products against present and forthcoming VOCs (virus variants of concern), potentially benefiting antibody-deficient patients. In this study, we compared the reactivity and neutralizing potential of commercial Ig products focusing on those produced during the period March 2021 to September 2022. Reactivity towards the Wuhan SARS-CoV-2 strain and nine later variant strains was assessed. The results shed light on not only the potential benefit, but also relative limitations, of current commercial IgRT batches against the evolving SARS-CoV-2 virus.

## Materials and Methods

### Immunoglobulin Batches

Seventy-four commercial Ig batches for clinical use were analyzed in the present study. The companies CSL Behring (King of Prussia, PA, USA; *n* = 29), Octapharma (Lachen, Switzerland; *n* = 2), and Takeda (Tokyo, Japan; *n* = 43) were represented. Aliquots from each respective batch were saved for analysis. Product Ig concentration ranged from 100 to 200 g/L, but before antibody analysis, the concentration of all samples was adjusted to 100 g/L by dilution with phosphate-buffered saline. The present study included all consecutive commercial immunoglobulin batches used from March 2021 to September 2022 (*n* = 52). A selection of older batches (*n* = 22), the earliest from September 2019, was also included as controls and to establish a pre-pandemic baseline level [7].

### Plasma Samples

The present study used additional plasma samples from two individuals with X-linked agammaglobulinemia (XLA) that were collected for a clinical trial previously described [8]. The use of these samples and data was approved by the Swedish Ethical Review Authority (ID2021-00,451). All participants provided written informed consent prior to inclusion in the trial.

### SARS-CoV-2 Antibody Assessment

Binding and pseudo‐neutralizing antibodies (ACE2 blocking) against SARS‐CoV‐2 wild type/Wuhan (nucleocapsid and spike), Alpha (B.1.1.7), Beta (B.1.351), Delta (B.1.617.2 AY.4), Omicron variants (BA.1, BA.1 + L452R, BA.1.1, BA2, BA.3), and IHU (B.1.640.2) were measured using the V‐PLEX SARS‐CoV‐2 10-plex panels 2 and 25 (Meso Scale Diagnostics, MD, USA) for IgG and ACE2 according to the manufacturer’s instruction at the SciLifeLab Affinity Proteomics Unit (Uppsala, Sweden). For binding antibody measurement, plasma samples were diluted 1:50,000 and detected using a SULFO-TAG conjugated mouse monoclonal anti-human IgG antibody. For inhibition of ACE2 spike binding, the samples were diluted 1:100 to allow competition with the recombinant human ACE2 conjugated with SULFO-TAG for binding to the spike antigen. The plates were analyzed on a MESO QuickPlex SQ 120 instrument, an electrochemiluminescence (ECL) reader measuring the light emitted from the SULFO-TAG. Results are reported in AU/ml and derived from back fitting the measured signals for samples to calibration curves generated for each plate.

## Results

**Fig. 1:**
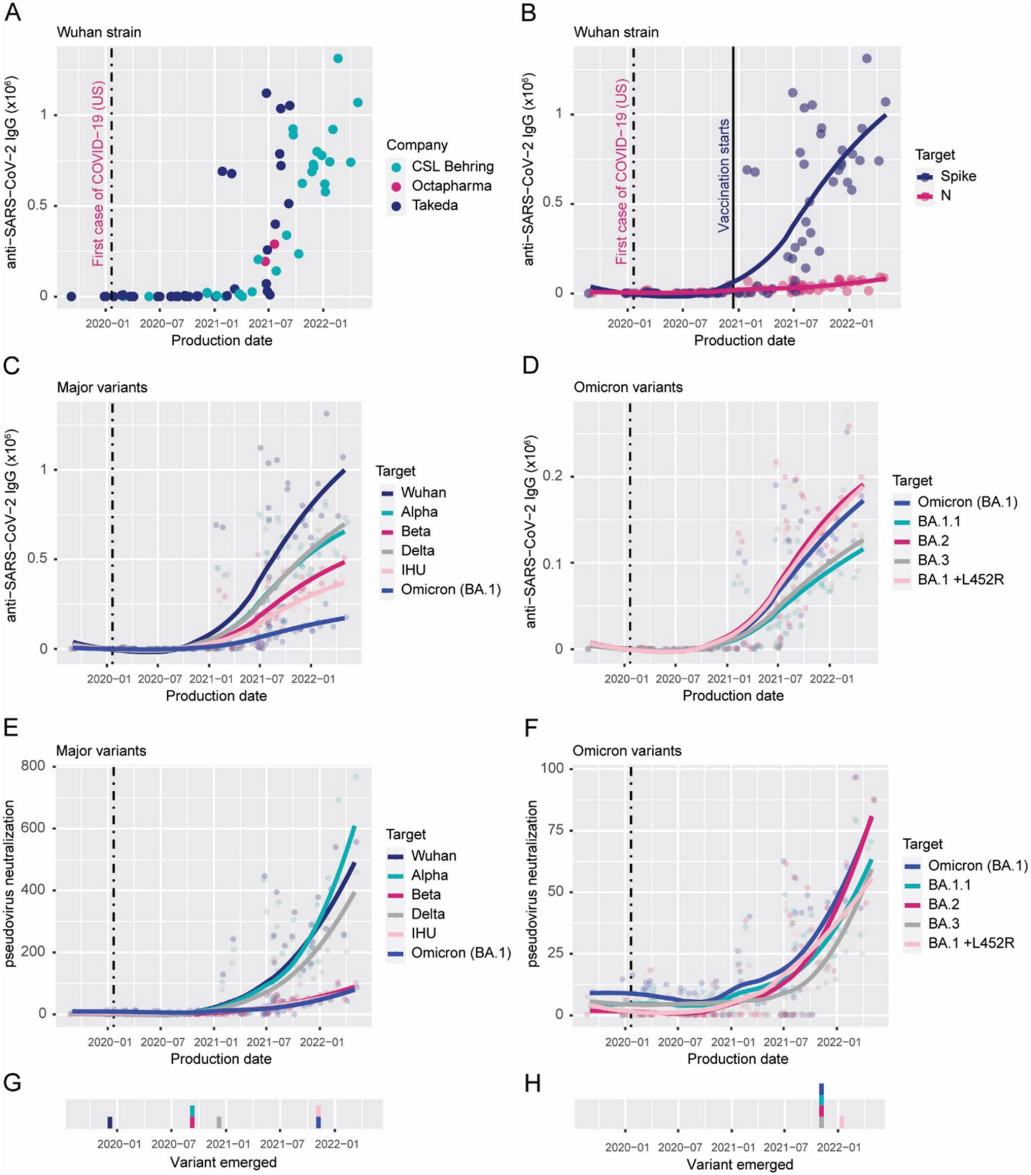
Vaccine-induced SARS-CoV-2 antibodies are consistently present in immunoglobulin preparations produced 18 months after the outbreak and later. **A** Anti-SARS-CoV-2 spike protein antibody quantity in batches of commercial immunoglobulin products used in clinical practice at the Immunodeficiency Unit at Karolinska University Hospital in relation to production date is plotted. The manufacturers are represented by different colors. **B** Antibody reactivity to the SARS-CoV-2 nucleocapsid (N) is specific for immunity after infection and rises slowly in comparison with the increase in antibody reactivity to the spike protein. Immunity in plasma donors is therefore mainly the result of vaccination. Antibody reactivity (**C**, **D**) and pseudovirus neutralization (**E**, **F**) to major virus variants and Omicron subvariants increase in parallel with reactivity to the original (Wuhan) strain. Lines were fitted using locally estimated scatterplot smoothing (LOESS) based on the individual immunoglobulin batch results (filled circles). Filled circles and lines are color-coded to show the results for each SARS-CoV-2 variant. Reactivity to certain VOCs is present in Ig batches produced before the virus variant was identified (**G**, **H**), implying that a degree of cross-reactivity exists between the tested variants. The virus variants are presented from top to bottom in the order they emerged (data from www.ecdc.europa.eu). A vertical dashed line indicates the first identified case of COVID-19 in the USA

Ig batches produced after July 2021 were consistently positive for SARS-CoV-2 antibodies to the Wuhan strain spike protein (Fig. 1A). This date is approximately 18 months after the first confirmed COVID-19 case and about 7 months after SARS-CoV-2 vaccination started in the USA, where most plasma donations occur. Antibodies directed at the SARS-CoV-2 nucleocapsid (N) are generated in response to natural infection but not vaccination. We plotted anti-spike and anti-N antibody values in relation to production time for all 74 batches (Fig. 1B). From July 2021, Ig batches contained donated plasma in which SARS-CoV-2 immunity is dominated by anti-spike antibodies and, therefore, essentially a result of vaccination.

Next, we tested the collected batches for IgG against the SARS-CoV-2 variants Alpha, Beta, Delta, and IHU as well as the Omicron subvariants BA.1, BA.1.1, BA.2, BA.3, and BA.1 with the L452R mutation. Paralleling the vaccine-induced increase in spike IgG against the Wuhan strain, proportionally lower levels of IgG to all these SARS-CoV-2 variants and subvariants were detected. In most cases, IgG reactivity was already present in Ig batches produced before the new virus strain was detected and must, therefore, largely be due to cross-reactivity of vaccine-induced antibodies. Nevertheless, to examine if IgG reactivity to any virus strains increased because of natural strain-specific infection, we assessed if the increase over time for any variant had a steeper slope than the Wuhan strain (Figure S1A, B). This was not the case, which also was visualized by fitting lines that depict the variant/Wuhan strain antibody ratio (Figure S1C, D), which was horizontal for all variants. Therefore, although the antibody reactivity to all SARS-CoV-2 variants increased over time, this is interpreted as a result of increased vaccine-induced antibodies in the Ig products that bind all tested variants to varying extents.

All IgG antibodies that bound to the respective spike protein variant were assessed (Fig. 1A–D), but only a subset of these antibodies was expected to interfere with virus entry into host cells. By only measuring the antibodies blocking the spike protein-ACE2 interaction, we estimated the quantity and/or quality of neutralizing antibodies to each SARS-CoV-2 strain (Fig. 1E, F). Similar to the binding antibodies, neutralizing antibodies increased over time for all tested virus strains.

**Fig. 2:**
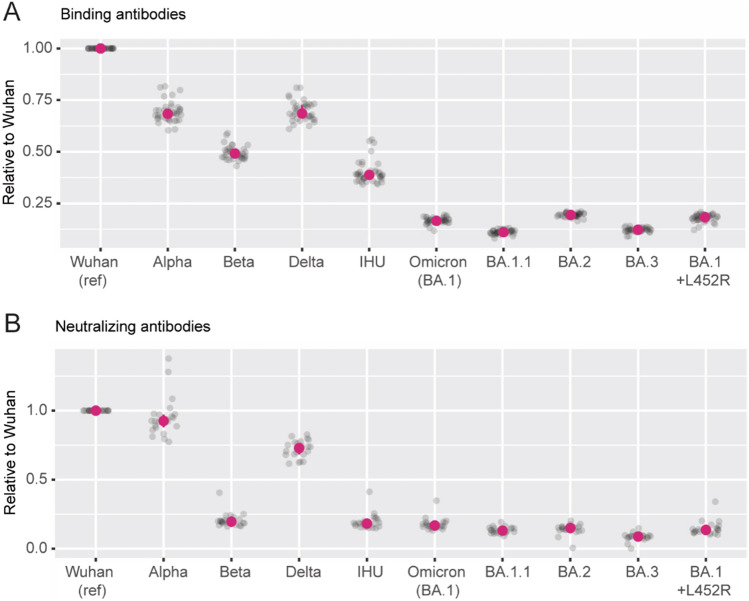
Vaccine-induced SARS-CoV-2 antibodies display consistent degrees of cross-reactivity to later virus variants. To assess the degree of cross-reactivity, samples that were unequivocally positive for the included targets were extracted and all results were expressed as a ratio with results for the Wuhan strain as the denominator. Results for **A** binding antibodies and **B** antibodies with neutralizing potential are shown. The virus variants are presented from left to right in the order they were first identified. Red dot represents the median and vertical lines the interquartile range, which in most cases is very small

After extracting only those batches with clearly positive reactivity for all tested SARS-CoV-2 variants, we plotted the variant/Wuhan strain antibody ratio for all nine tested variants to define their relative cross-reactivity (Fig. 2A). Reactivity decreased proportionally with the date the variant strain was detected, with some exceptions. Antibody reactivity to the Delta variant was stronger than to the earlier variant Beta. Similarly, the IHU-variant reactivity was more substantial than Omicron BA.1 despite appearing approximately simultaneously. Regarding the included Omicron variants, which all were detected in a relatively short period (Table S1), small but consistent differences could be detected with the lowest antibody binding to the BA.1.1 and BA.3 variants.

The Ig batches’ relative potential to neutralize the Alpha variant was nearly as good as for the Wuhan strain, but the potential to neutralize the Beta variant was markedly lower (Fig. 2B). Similar to the binding antibodies (Fig. 2A), neutralizing antibody responses for the Delta variant were higher than for the Beta variant and all subsequent Omicron VOCs. However, the prominent binding to the IHU spike protein was not reflected in a similarly elevated neutralizing potential.

**Fig. 3:**
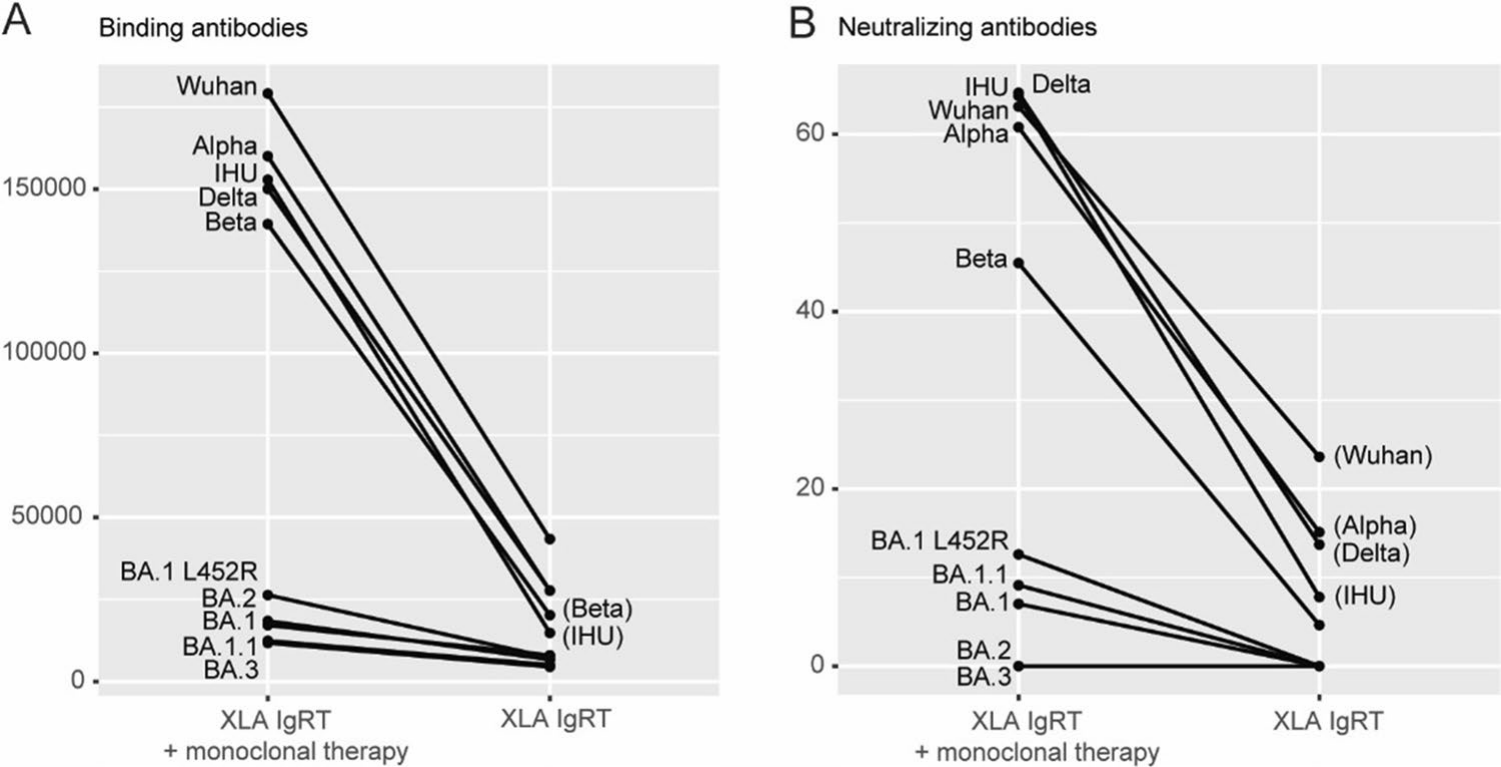
SARS-CoV-2 variant reactivity in plasma samples from XLA patients with immunoglobulin replacement therapy. Plasma was sampled from two X-linked agammaglobulinemia (XLA) patients that have no endogenous antibody production but were on continuous immunoglobulin replacement therapy (IgRT). One patient had also received treatment with monoclonal SARS-CoV-2 therapy (sotrovimab) approximately 1 month prior to blood sampling. All binding antibodies (**A**) and only neutralizing antibodies (**B**) towards ten SARS-CoV-2 variants were quantified

Finally, to confirm that these results are translated to patients receiving the Ig products, we sampled two X-linked agammaglobulinemia (XLA) patients with no endogenous antibody production and receiving regular subcutaneous IgRT at our outpatient clinic in Stockholm. They were sampled on the same day in March 2022. Interestingly, one of these patients had received monoclonal SARS-CoV-2 antibody treatment with sotrovimab before sampling. Spike-binding antibodies (Fig. 3A) and neutralizing antibodies (Fig. 3B) were quantified using the same methodology in plasma from these two patients. The patterns of cross-reactivity in these plasma samples were largely consistent with those for the Ig batches except for the unexpectedly high amounts of binding and neutralizing antibodies towards the IHU variant in the patient that also received sotrovimab treatment. As expected, the Omicron variants were universally distinguished by low binding and neutralizing capacity.

## Discussion

Transmission and disease severity of SARS-CoV-2 have been profoundly diminished by the widespread use of COVID-19 vaccines, which were developed to neutralize the original strain of the virus that started spreading in Wuhan, China, in late 2019. With a delay of about 6 months after vaccination started, vaccine-induced antibodies began to be detected in commercially available donor plasma-derived Ig products used to control infections in patients with antibody deficiency. However, the emergence of new SARS-CoV-2 variants as Omicron which evade immunity because of multiple mutations in the spike protein raises concerns about the efficacy of these Ig products against COVID-19 in patients with antibody deficiencies. Here, we examined how well vaccine-induced SARS-CoV-2 antibodies in off-the-shelf Ig products interact with the spike protein of nine VOCs and observed lower binding and neutralization potential in all of them compared to the original Wuhan strain.

The Alpha variant was detected in the UK in September 2020 and quickly became the dominating variant in several countries (Table S1). The Beta variant emerged in South Africa simultaneously, but its spread was more limited. Consistent with our data, laboratory investigations and real-world data suggest that cross-reactivity to the Alpha variant is less hampered than the Beta variant [9, 10]. The Ig batches neutralized the Delta variant relatively efficiently, consistent with observations that breakthrough infection with this strain is less likely in vaccinated individuals with high levels of antibodies. In contrast, antibody levels do not correlate with breakthrough infection with the Omicron strain [11]. Moreover, although the IHU variant was detected simultaneously as the first Omicron variant, it contains fewer spike protein mutations and only spread locally in France. Consistently, we observed relatively strong antibody reactivity against the IHU variant spike protein [12]. When it first emerged, the IHU variant attracted attention, but the World Health Organization has not classified it as VOC (www.who.int).

Previous studies have concluded that Omicron variants BA.1, BA.2, and BA.3 evade vaccine-induced antibody neutralization with comparable high-efficiency [13], which is consistent with our data. Similarly, plasma industry representatives have reported a dramatic loss of neutralization potential against Omicron with their products, on average 20-fold lower than against the Wuhan variant [14]. The third lineage of Omicron, BA.3, has introduced no new mutations in the spike protein but includes a subset of mutations from both BA.1 and BA.2 [15]. Both regarding all binding antibodies and antibodies with neutralizing potential in the Ig batches, reactivity against BA.3 was slightly lower than for BA.1 and BA.2. The spike protein mutation L452R emerged with the Delta variant and its absence in the BA.1 Omicron variant has been suggested as an explanation for the lower pathogenicity in the latter strain [16]. BA.1 eventually attained the L452R mutation, which is included in the later subvariants BA.4 and BA.5 [17], but the feared increase in pathogenicity did not occur [18]. According to our data, the L452R mutation does not significantly alter antibody binding or neutralization potential. More recently, Omicron-specific and bivalent vaccines have been shown to be safe and effective [19]. Whether this will influence the antibody content in future Ig batches primarily depends on to what extent the plasma donor population will receive the vaccines.

Patients with XLA do not produce any endogenous antibodies and thus serve as an excellent model to study the role of IgRT in SARS-CoV-2 immunity. We have previously shown that these individuals obtain a potent T cell–mediated immunity after vaccination, which most likely protects them from severe disease [20]. However, it also appears that antibodies are needed to clear the virus from mucosal surfaces [21]. Thus, since commercial Ig preparations do not contain sufficient neutralizing antibody levels against emerging SARS CoV-2 variants, XLA patients could potentially be long-term carriers of the virus. There have been a few case reports from the pre-vaccine era describing complicated cases of COVID-19 in patients with XLA [22, 23]. However, we are unaware of long-term viral carriage or shedding in fully vaccinated XLA patients. This suggests that T cells, potentially together with IgRT, effectively clear the virus from mucosal surfaces.

Overall, passive immunity obtained by IgRT reflects vaccine-induced antibodies in the plasma donor population with a production delay of several months. The Ig batches demonstrated variable levels of cross-reactivity against all tested spike protein variants, with limited capacity to neutralize Omicron variants.

## Supplementary Information

Below is the link to the electronic supplementary material.

Supplementary file1 (DOCX 223 KB)

## Data Availability

Data availability is specified in the original study [8].
