# Supplementary material for: SARS-CoV-2 Antibodies in Commercial Immunoglobulin Products Show Markedly Reduced Cross-reactivities Against Omicron Variants
Source: J Clin Immunol. 2023 Apr 14;43(6):1075–82. doi: 10.1007/s10875-023-01486-8 (PMC10102687; doi:10.1007/s10875-023-01486-8)
Supplement: Supplementary file 1 — Supplementary file1 (DOCX 223 KB) [file 10875_2023_1486_MOESM1_ESM.docx]

# Supplementary Information


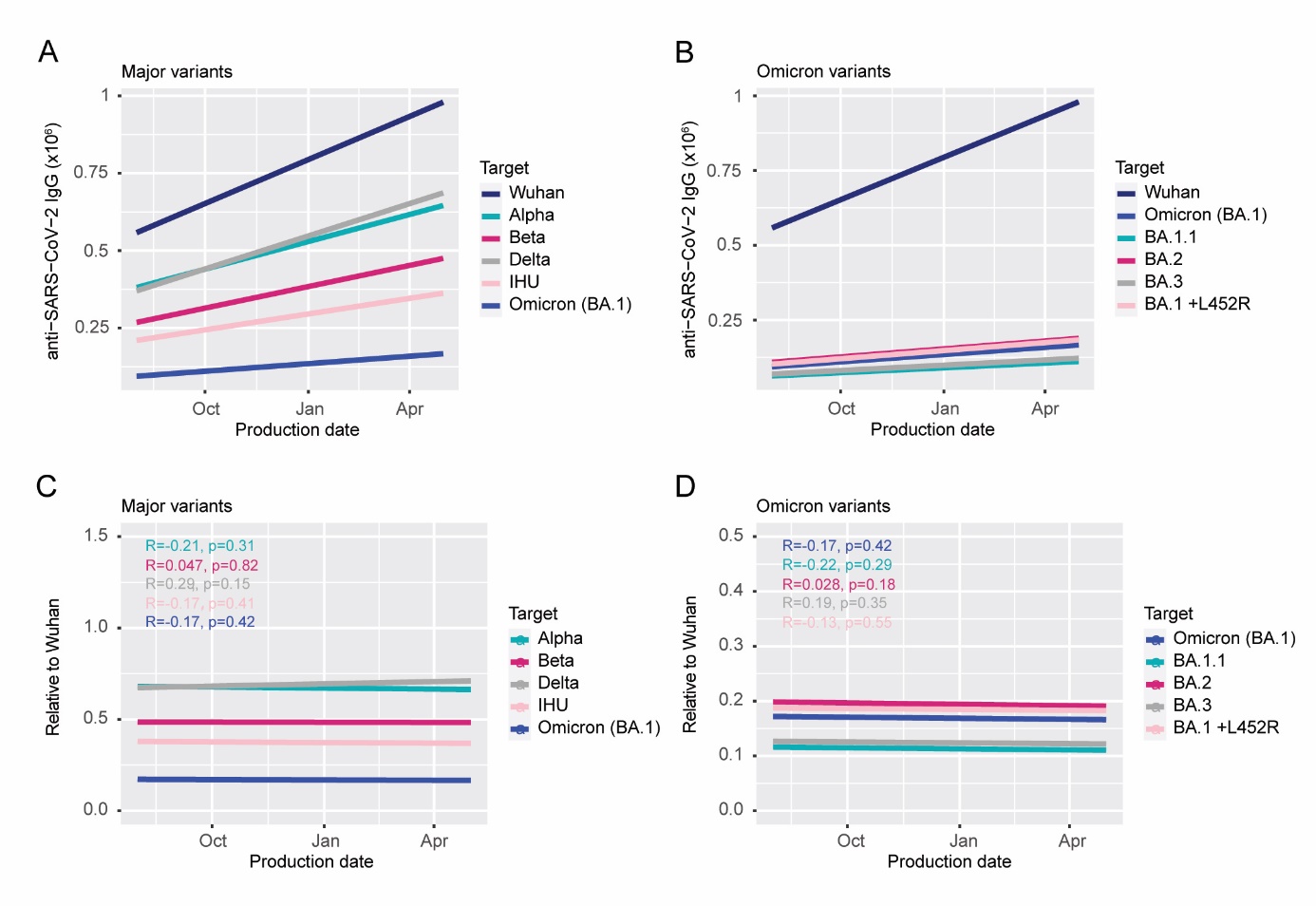


Figure S1. **Antibody reactivity to SARS-CoV2 variants and the original Wuhan strain increase similarly.** Ig batches produced after July 2021 had to a large extent positive results for all tested virus strains. A straight line was fitted to each strain to visualize the increase in antibody reactivity over time (A, B) and the variant/Wuhan strain ratio was similarly plotted (C, D) to demonstrate that the reactivity proportionally to the Wuhan strain (vaccine-induced) reactivity is constant from July 2021 to May 2022. Linear correlation was assessed using Pearson correlation coefficient.

| **Table S1. Nine SARS-CoV-2 variants and associated spike protein mutations** | | |
| --- | --- | --- |
| **SARS-CoV-2 variant** | **First detected** | **Spike protein mutations** |
| Alpha (B.1.1.7) | Sep 2020 | 69del, 70del, 144del, N501Y, A570D, D614G, P681H, T716I, S982A, D1118H |
| Beta (B.1.351) | Sep 2020 | L18F, D80A, D215G, 241del, 243del, K417N, E484K, N501Y, D614G, A701V |
| Delta (B.1.617.2) | Dec 2020 | T19R, G142D, 156del, 157del, R158G, L452R, T478K, D614G, P681R, D950N |
| B.1.640.2 (IHU) | Nov 2021 | P9L, E96Q, 136del, 137del, 138del, 139del, 140del, 141del, 142del, 143del, 144del, R190S, D215H, R346S, N394S, Y449N, E484K, F490S, N501Y, D614G, P681H, T859N, D1139H |
| Omicron (BA.1) | Nov 2021 | A67V, 69del, 70del, T95I, 142del, 142del, 144del, 145del, N211I, L212del, ins214EPE, G339D, S371L, S373P, S375F, K417N, N440K, G446S, S477N, T478K, E484A, Q493R, G496S, Q498R, N501Y, Y505H, T547K, D614G, H655Y, N679K, P681H, N764K, D796Y, N856K, Q954H, N969K, L981F |
| Omicron (BA.1.1) | Nov 2021 | BA.1 mutations with an additional R346K mutation |
| Omicron (BA.2) | Nov 2021 | T19I, LPPA24-27S, G142D, V213G, G339D, S371F, S373P, S375F, T376A, D405N, R408S, K417N, N440K, S477N, T478K, E484A, Q493R, Q498R, N501Y, Y505H, D614G, H655Y, N679K, P681H, N764K, D796Y, Q954H, N969K |
| Omicron (BA.3) | Nov 2021 | A67V, 69-70del, T95I, G142D, 143del, 144del, 145del, N211I, 212del, G339D, S373P, S375F, D405N, K417N, N440K, G446S, S477N, T478K, E484A, Q493R, N501Y, Y505H, D614G, H655Y, N679K, P681H, N764K, D796Y, Q954H, N969K |
| Omicron (BA.1+L452R) | Jan 2022 | BA.1 mutations with an additional L452R mutation |
| Data from www.ecdc.europa.eu. | | |
